# Investigation of dynamic microbial migration patterns in the respiratory tract

**DOI:** 10.3389/fcimb.2025.1542562

**Published:** 2025-04-22

**Authors:** Ping Fang, Yanhua Wen, Wenjing Deng, Ruobing Liang, Ping He, Chunya Wang, Na Fan, Kaikai Huo, Kaikai Zhao, Cong Li, Ying Bai, Yuwan Ma, Long Hu, Yuanlin Guan, Shuanying Yang

**Affiliations:** ^1^ Department of Pulmonary and Critical Care Medicine, The Second Affiliated Hospital of Xi’an Jiaotong University, Xi’an, China; ^2^ Department of Scientific Affairs, Hugobiotech Co., Ltd., Beijing, China

**Keywords:** lower respiratory tract infections, airway microbiome, source tracking analysis, pathogen migration, metagenomic next-generation sequencing (mNGS)

## Abstract

**Background:**

The role of the respiratory microbiome in lung diseases is increasingly recognized, with the potential migration of respiratory pathogens being a significant clinical consideration. Despite its importance, evidence elucidating this phenomenon remains scarce.

**Methods:**

This prospective study collected clinical samples from patients with suspected lower respiratory tract infections (LRTI), including oropharyngeal swabs (OPS), sputum, and bronchoalveolar lavage fluid (BALF). Metagenomic next-generation sequencing (mNGS) was employed to analyze respiratory microbial diversity, complemented by Bayesian source tracking and sequence alignment analyses to explore pathogen migration patterns.

**Results:**

A cohort of 68 patients was enrolled, with 56 diagnosed with LRTI and 12 with non-infectious respiratory conditions. A statistically significant disparity in respiratory microbiome diversity was observed between infected and non-infected groups (*p* < 0.05). Intriguingly, no significant variations in microbial community structure, including alpha and beta diversity, were detected across different respiratory tract sites within individuals. The Bayesian source tracking analysis revealed a pronounced migration pattern among pathogens compared to the overall microbial community, with migration ratios of 51.54% and 1.92%, respectively (*p* < 0.05). Sequence similarity analysis further corroborated these findings, highlighting a notable homology among specific migrating pathogens.

**Conclusion:**

This study represents a pioneering effort in deducing pathogen migration patterns through microbial source tracking analysis. The findings provide novel insights that could significantly advance clinical diagnostics and therapeutic strategies for respiratory infections.

## Background

The respiratory system constitutes a complex and dynamic microecosystem, characterized by a continuous flux of microbial migration and clearance processes (Dickson et al., 2016). This dynamic interplay results in a variable bacterial burden and microbiome composition, challenging the notion of a static respiratory microbiota (Fabbrizzi et al., 2019). Anatomically, the respiratory system is divided into the upper respiratory tract (URT) and lower respiratory tract (LRT), each harboring distinct microbial communities yet interconnected through microbial migration, a common physiological phenomenon (Man et al., 2017; Natalini et al., 2023). The URT, encompassing the nasal cavity, paranasal sinuses, and oropharynx, interfaces directly with the external environment, making it susceptible to microbial colonization. In contrast, the LRT, comprising the trachea and lungs, must maintain a delicate balance of microbial presence to preserve optimal gas exchange (Natalini et al., 2023). The migration of microbes within the respiratory tract is a complex process that likely plays a critical role in the etiology and progression of respiratory diseases.

While microbial migration is a biologically plausible phenomenon with physiological underpinnings, concrete evidence linking this process to health outcomes and disease pathogenesis is scarce. Despite advancements in metagenomic next-generation sequencing (mNGS) technology (Chiu and Miller, 2019; Lin et al., 2023), which has enabled a more nuanced understanding of the respiratory microbiota, research has predominantly focused on the differences in microbial composition across various patient populations, rather than the dynamic process of microbial movement between different regions of the respiratory tract (Man et al., 2017; Lin et al., 2023). To provide insights into the mechanisms of respiratory infections, we investigate the migration of pathogens in the context of lower respiratory tract infections (LRTIs). LRTIs encompass a collection of infections affecting the respiratory tract below the larynx and have consistently posed a significant public health concern, linked to significant morbidity and mortality (Diseases and Injuries, 2020).

To achieve our objectives, we have prospectively examined patients with suspected LRTIs. Utilizing mNGS, we collected clinical samples from various respiratory tract site, including bronchoalveolar lavage fluid (BALF), sputum, and oropharyngeal swabs (OPS). Through a comprehensive analysis of microbial diversity, Bayesian source tracing, and sequence alignment, we sought to elucidate the migration patterns of pathogens in LRTIs and their implications for respiratory health.

## Materials and methods

### Ethics

This study was approved by the Clinical Research Ethics Committee of the second affiliated hospital of Xi’an Jiaotong University, and informed written consent was obtained from all participants.

### Clinical information collection

We prospectively recruited 79 suspected LTRI patients admitted to Department of Pulmonary and Critical Care Medicine of The Second Affiliated Hospital of Xi’an Jiaotong University between July 2022 to July 2023. The age, gender, medical history, clinical symptoms, lung CT results, blood routine, biochemical inflammatory markers, immune function test results, clinical diagnosis information, medication details, duration of mechanical ventilation, ICU stay, hospital stay, and other relevant information were collected from all patients.

### Sample collection

Out of 79 patients, 11 were excluded due to the absence of BALF samples. The remaining 68 patients provided a total of 204 samples, encompassing three types: OPS, sputum, and BALF. The OPS was collected by swabbing the soft palate, the root of the tongue and the left and right lateral walls of the oropharyngeal cavity, twice at each surface, using a sterile absorbent swab. Sputum was obtained through natural expectoration or using a disposable sputum suction catheter. BALF was collected by fiber-optic bronchoscopy from participants. All the samples were immediately sent for laboratory tests under -20°C.

### Identification of causative pathogens

Besides mNGS, conventional tests were also performed for diagnosis, including smear microscopy, culture, PCR detection, and antigen/antibody immunology. In addition, the 1,3-β-d glucan test, galactomannan antigen detection, and T cell spot test for tuberculosis were performed as appropriate. Utilizing comprehensive data derived from mNGS, conventional diagnostic assays, and thorough clinical evaluations, the causative pathogens were identified by a multidisciplinary panel of experts, including intensivist, clinical microbiologists, and bioinformaticians with extensive experience in mNGS.

### Metagenomic sequencing and data analysis

OPS, sputum and BALF samples were sent for next generation sequencing (Hugobiotech, Beijing, China). QIAamp DNA Micro Kit (QIAGEN, Germany) was applied to extract DNA following the manufacturer’s instructions. DNA libraries were then constructed using QIAseq™ Ultralow Input Library Kit for Illumina (QIAGEN, Germany) according to its manual. All constructed libraries were quality assessed by Qubit (Thermo Fisher, USA) and Agilent 2100 Bioanalyzer (Agilent Technologies, USA). The qualified DNA libraries were finally sequenced on Illumina Novaseq 6000 platform (Illumina, USA) with an expected average sequencing volume of ~ 25 million reads per sample.

Raw data were trimmed adapter sequences and filtered low-quality reads by Trimmomatic (v0.39) (Bolger et al., 2014), and clean reads were then mapped to human reference genome GRCh38.p13 to remove host reads by bowtie2 (v2.4.2) (Langmead and Salzberg, 2012) based on kneaddata (v0.7.4) (https://github.com/biobakery/biobakery/wiki/kneaddata). The non-human data were classified by simultaneous alignment to the reference microbial sequences from archaea, bacteria, viruses, and fungi, which were obtained from the NCBI Nucleotide database (2020-12-21). Sequence alignment was performed by BLASTN (v2.11.0+) with “megablast” option, and only reads uniquely aligning to microbial taxa were tallied. Functional analysis of microbiome data was conducted using HUMAnN3 to identify differences in microbial metabolic potential between infected and non-infected patients, as well as across different sample types (Beghini et al., 2021).

### Microbial migration analysis using Bayesian source tracking method

SourceTracker (v1.0.1) is a Bayesian-based approach designed to estimate the proportion of microbial communities in an unknown experimental sample (referred to as the “sink”) that originate from user-defined source samples (Knights et al., 2011). SourceTracker calculates possibility scores for each species detected in the sink sample, a species was considered to originate from a source sample if its possibility score in the source was higher than that in the sink sample. In this study, we conducted source tracking analysis to investigate microbial migration patterns among OPS, sputum, and BALF samples from the same patient. If microbial species detected in the three sample types from the same patient simultaneously satisfied the following three migration patterns: OPS originating from sputum, OPS originating from BALF, and sputum originating from BALF, they were considered to exhibit LRT-to-URT dynamics (migration from the lower respiratory tract to the upper respiratory tract). Conversely, they were considered to exhibit URT-to-LRT dynamics.

### Microbial migration analysis using sequence alignment method

To ensure robust pathogen migration analysis via sequence alignment, only samples with sufficient pathogen reads were considered. Samples from patients with at least 1,000 reads of a single common pathogen across all three sample types were retained; otherwise, they were excluded. The reads corresponding to the single common pathogen in each retained sample were assembled using Megahit software (v1.2.9) (Li et al., 2015). The assembled genomic sequences underwent redundancy removal using CD-HIT (v4.8.1) with parameters set to -c 0.99 and -l 10 (Fu et al., 2012). Contigs were aligned to reference genomes using minimap2 (v2.17) (Li, 2018). SNPs between samples and reference genomes were identified using samtools (v1.10) “mpileup” (Li et al., 2009). SNPs were filtered by excluding sites identical across all samples and those missing in over one-third of the samples. Subsequently, pairwise SNP similarity between samples was computed as the ratio of consistent sites (identical nucleotide sequences) to shared sites (present nucleotide sequences). Pairwise SNP similarity ranged from 0 to 1.

### Statistical analysis

For each sample, alpha diversity metrics (Shannon diversity, Simpson diversity, Chao 1 and ACE diversity index) were computed using R library “vegan”. The matrix of β-diversity distances between samples was calculated using Bray-Curtis distance metrics and subsequently analyzed through principal coordinates analysis (PCoA) by R library “ape” (v5.5). The Chi-square test was used to evaluate the statistical significance of differences between groups for categorical variables. For comparisons between infection and non-infection microbiomes, the Wilcoxon rank-sum test was applied. Additionally, differential pathway analysis was performed using STAMP, with statistical significance assessed through the Kruskaldance,E H test and Wilcoxon rank-sum test. Data analyses were performed in R version 4.1.0 software. A *p* value < 0.05 was considered significant.

## Results

### Patients and sample characteristics

A total of 68 patients were enrolled in this study, including 56 finally diagnosed with LRTI and 12 with non-infectious respiratory diseases. The cohort included 47 males (69.1%) and 21 (30.9%) females, with a median age of 68 years (IQR, 54-74 years). A comprehensive overview of the clinical characteristics of these individuals was presented in **Table 1**. Among the enrolled patients, 63.2% (43/68) presented with cough, a common symptom of respiratory infections. Additionally, the majority of patients had pre-existing medical conditions, such as hypertension, hypoproteinemia, and kidney disease. Laboratory assessments indicated elevated mean plasma levels of C-reactive protein at 73.15 mg/L (IQR, 12.93-114.50 mg/L) and procalcitonin at 0.243 ng/mL (IQR, 0.059-0.918 ng/mL). Among the 12 patients with non-infectious respiratory diseases, 11 were diagnosed with interstitial pneumonia, and one was diagnosed with lung cancer.

**Table 1:** Clinical characteristics of the 68 suspected LRTI patients.

Demographic characteristics	Value
Age (years) median, IQR	68 [57, 74]
Sex	
Female	21 (30.9%)
Male	47 (69.1%)
Underlying disease	
Diabetes	11 (16.2%)
Hypertension	23 (33.8%)
Hypoproteinemia	15 (22.1%)
Kidney disease	9 (13.2%)
Liver disease	8 (11.8%)
Clinical symptoms	
Fever	34 (50%)
Cough	43 (63.2%)
Expectoration	31 (45.6%)
Shortness of breath	38 (55.9%)
White cell count (10^9^ cells/L) median, IQR	7.62 [5.63, 12.39]
C-reactive protein (mg/L) median, IQR	73.15 [12.93, 114.50]
Procalcitonin (ng/mL) median, IQR	0.243 [0.059, 0.918]
Critical patients	54 (79.4%)
Hospitalization duration (days) median, IQR	18 [13, 25]

LRTI, lower respiratory tract infections; IQR, interquartile range.

### Microbial diversity analysis revealed high similarity across respiratory tract sites

The mNGS approach generated an average of 24.68 million reads per sample (**Supplementary Table S1**), which is sufficient for robust microbial community analysis and downstream statistical comparisons.

**Figure 1:**
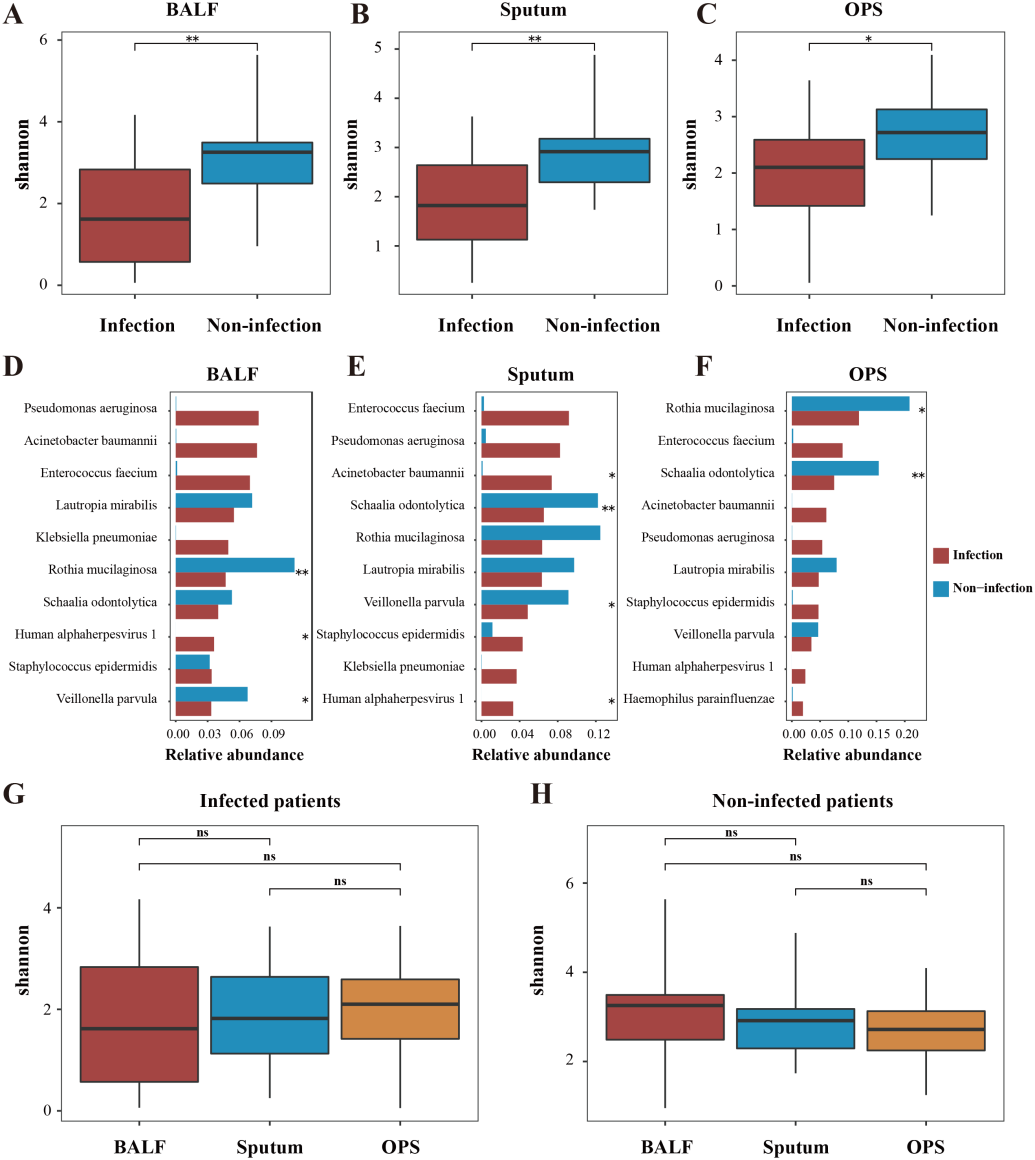
Alpha diversity variations in airway microbiome. Alpha diversity (Shannon index) comparisons were conducted between patients with infection (red) and those without (blue) in BALF **(A)**, sputum **(B)**, and OPS **(C)** samples. The top 10 abundant microbes in infected patients and their corresponding abundance in infected and non-infected patients were compared in BALF **(D)**, sputum **(E)** and OPS **(F)** samples. Comparisons of alpha diversity among three sample types for same individuals, stratified by infection status, were shown in G and **(H)** Significant differences are denoted by asterisk markers (The symbols * and ** represent *p* < 0.05 and <0.01, respectively). "ns" represents p > 0.05.

We first compared the LRT microbiome between LRTI patients and those with non-infectious conditions using BALF samples. The LRTI group exhibited a significantly lower Shannon index compared to the non-infectious group, (**Figure 1A**; **Supplementary Figure S1**), indicating reduced microbial diversity in the presence of infection. Furthermore, distinct patterns in the most abundant microbial taxa (top 10) were observed between the two groups (**Figure 1D**). Specifically, *Pseudomonas aeruginosa* emerged as the most abundant genus in BALF samples from LTRIs patients, while *Rothia mucilaginosa* predominated in the non-infectious group, which was in concordance with previous research (Man et al., 2017; Whiteside et al., 2021; Lin et al., 2023).

Interestingly, similar patterns were observed in sputum and OPS samples (**Figures 1B, C, E, F**; **Supplementary Figure S1**), with the latter representing a typical URT sample. These findings diverged from our initial expectations, as LRTIs are typically associated with the LRT rather than the URT. However, the URT microbiome also appeared to be influenced in infected patients, suggesting a broader impact of LRTIs on the respiratory ecosystem. We hypothesized that these observations might be attributed to the dynamic equilibrium of the airway microbiome.

**Figure 2:**
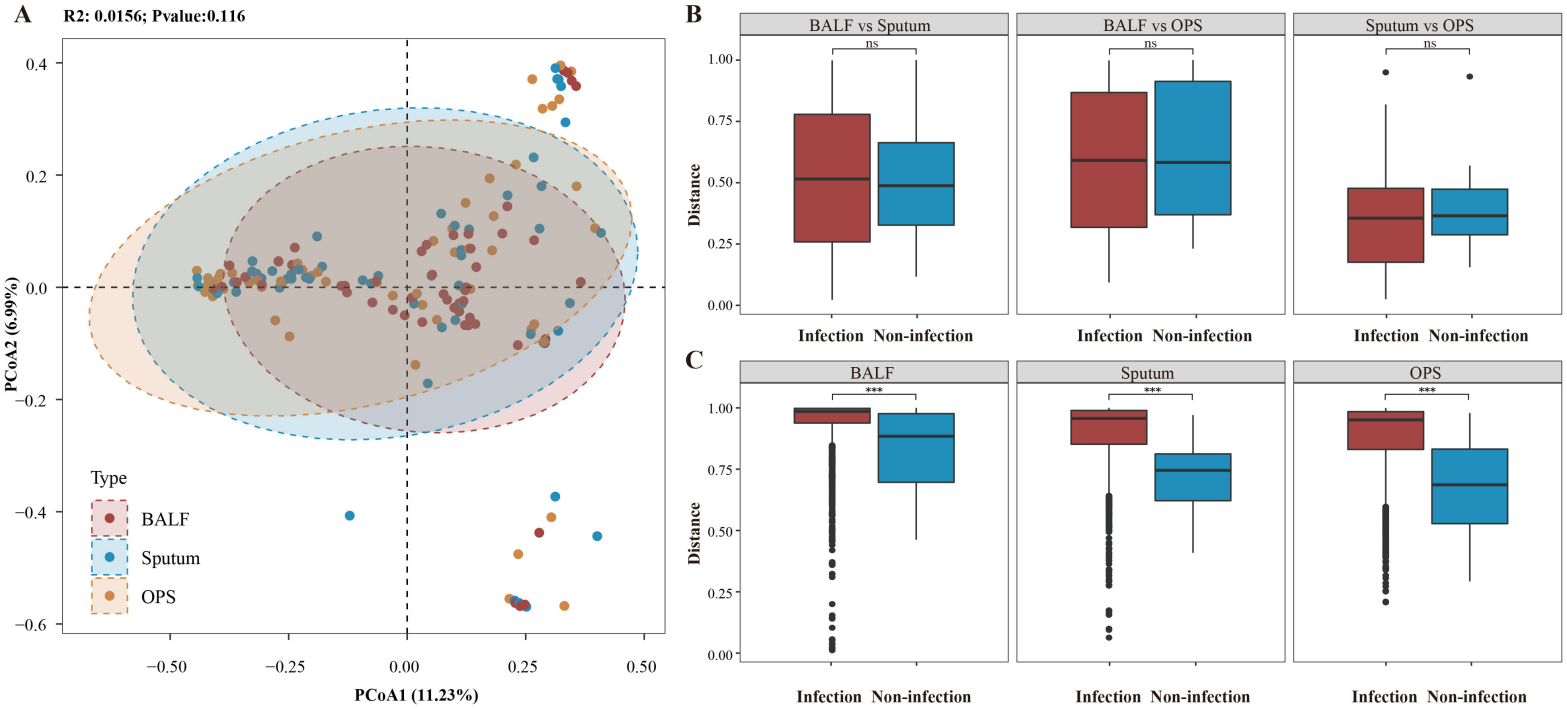
Beta diversity variations in airway microbiome. **(A)** Principal Coordinate Analysis (PCoA) of the three sample types for patients with infection, with the X and Y axes representing the first and second principal components, respectively. **(B)** Pairwise Bray-Curtis distance calculations between different sample types for the same individual, comparing distances for patients with and without infection. **(C)** Pairwise Bray-Curtis distance calculations within the same sample type among patients, comparing distances for patients with and without infection. Significant differences are highlighted with asterisk markers. *** represents p < 0.001, "ns" represents p > 0.05.

To explore this, we conducted a comprehensive multi-site microbiome comparison. Consistent with our hypothesis, no significant differences in microbial diversity were observed across BALF, sputum, and OPS samples in both LRTI patients (**Figure 1G** and **Supplementary Figure S2**) and non-infected individuals (**Figure 1H**; **Supplementary Figure S2**). Further beta diversity analysis using Principal Coordinate Analysis (PCoA) revealed no significant differences among the three sample types in either group (*p* > 0.05) (**Figure 2A**), indicating high similarity in microbial community structure across respiratory sites. Additionally, intra-individual analysis demonstrated consistent microbial communities across sampling sites, regardless of infection status (**Figure 2B**; **Supplementary Figure S3**), highlighting the stability of the respiratory microbiome within individuals. However, significant differences were observed between infected and non-infected patients, extending beyond BALF to include sputum and OPS samples.

To further explore the metabolic potential of microbial communities, we performed functional analysis to annotate genes and pathways in the metagenomic data. Significant differences were observed between infected and non-infected patients, as well as across different sample types (**Supplementary Figures S4**, **S5**). The results provide additional insights into the metabolic adaptations and pathogenic potential of microbial communities in respiratory infections.

Based on these findings, we hypothesize that respiratory infections may have broader systemic effects than previously recognized. Specifically, infection may not only alter the microbiome at the directly affected site but also influence seemingly unaffected regions, such as the URT, through the dynamic equilibrium of the respiratory tract. This suggests a complex interplay between localized infection and the overall respiratory microbiome, with potential implications for understanding the systemic effects of respiratory infections.

### Migration along respiratory tract revealed by source tracking analysis

**Figure 3:**
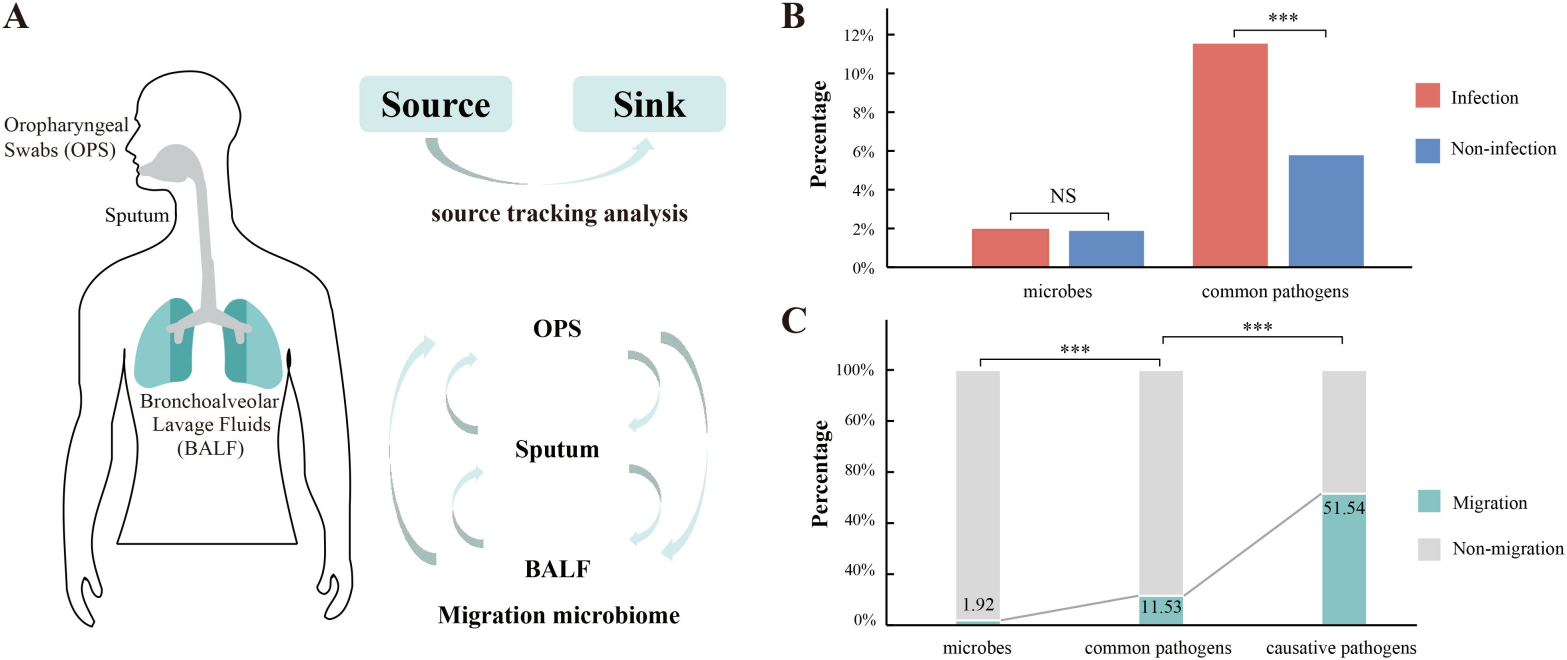
Source tracking analysis of airway microbes. **(A)** Schematic representation of the application of source tracking analysis to elucidate the migration patterns of airway microorganisms. **(B)** Comparative analysis of the percentage of migrated microorganisms within the entire microbiome and common pathogens between patients with and without infection. **(C)** Percentage distribution of migrated (cyan) versus non-migrated (grey) microorganisms across the entire microbiome, common pathogens, and causative pathogens. Significant variations are indicated by asterisk markers. *** represents p < 0.001, "ns" represents p > 0.05.

In this study, we identified a total of 5,461 microbial species across all patients, with the fewest species detected in BALF (2,688), followed by sputum (4,378) and OPS (4,923). Since many species were detected in multiple patients, we performed source tracking analysis on a total of 79,835 microbial taxa from the respiratory tracts of 68 patients (**Supplementary Figure S6**), including 5,330 taxa (6.7%) shared across all three sample types (**Figure 3A**). Among these, 58,719 taxa were from infected patients, and 21,116 were from non-infected patients. In infected patients, 1.92% (1,129/58,719) of microbial taxa exhibited migration, a proportion similar to that observed in non-infected patients (1.88%, 396/21,116) (**Figure 3B**). However, when focusing on common pathogens of all detected microbial taxa, the migration rate was significantly higher. Among the 1,474 common pathogens detected in infected patients, 11.53% (170/1,474) showed evidence of migration (**Figure 3C**), while in non-infected patients, 5.78% (19/329) of common pathogens exhibited migration. This indicates that common pathogens are more prone to migration than non-pathogenic microbes, and the proportion of migrating common pathogens was significantly higher in infected patients compared to non-infected patients (*p* < 0.01). Additionally, 97 out of the 1,474 common pathogens were clinically confirmed as causative agents, 51.55% (50/97) exhibited migration, highlighting the potential role of microbial migration in the pathogenesis of respiratory infections. Furthermore, >80% of the migrated causative pathogens exhibited bidirectional migration patterns.

### Individual level pathogen migration pattern

**Figure 4:**
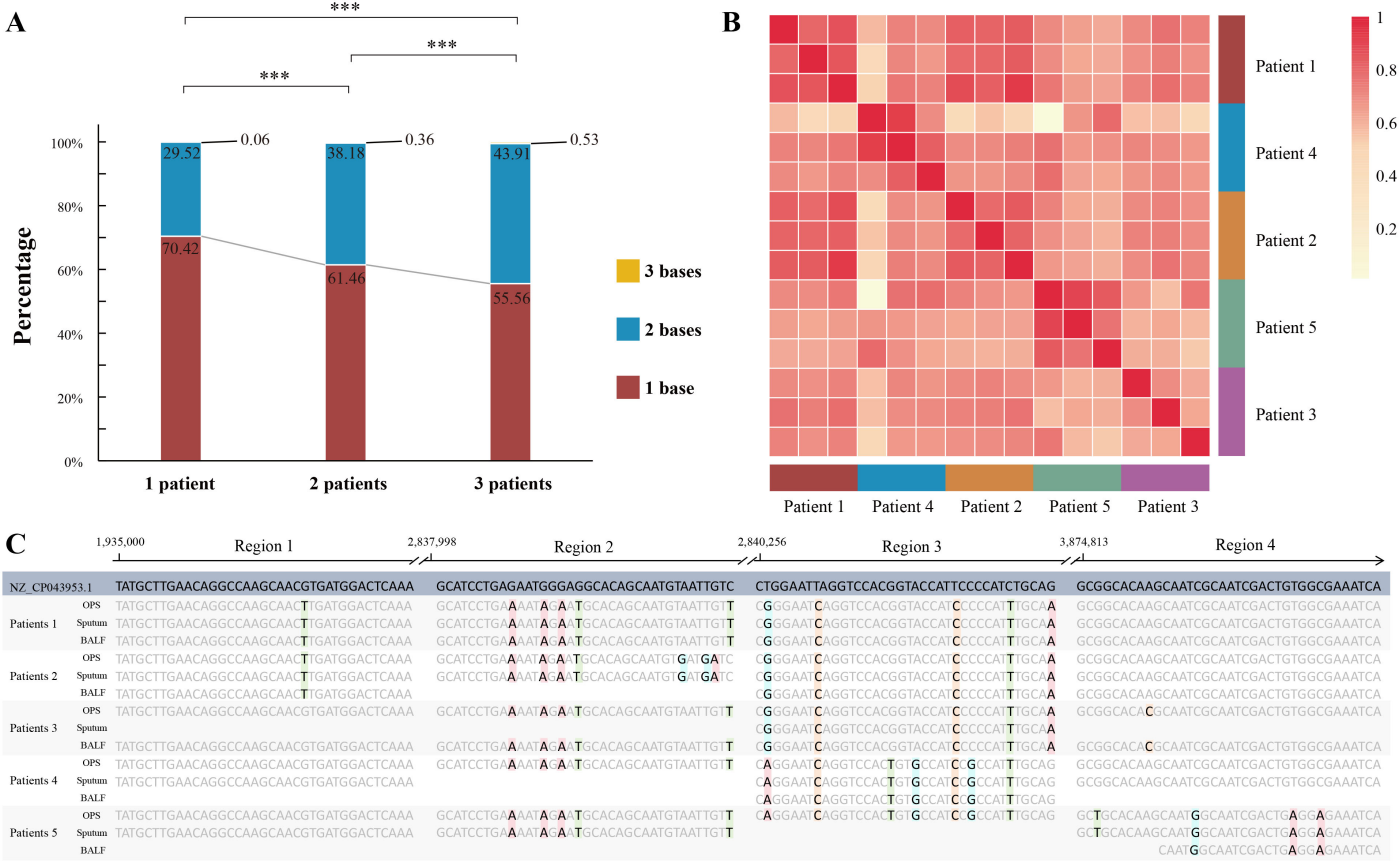
Sequence alignment analysis of migrated pathogens. **(A)** Stacked barplot illustrating the distribution of identical and differing bases when comparing samples from the same patient, two different patients, or three different patients. Significant variations are denoted by asterisk markers. **(B)** Heatmap representation of pairwise sample similarity for patients with *A baumannii* infection, with darker red indicating higher similarity (Methods section for details). **(C)** Multiple sequence alignment of contigs derived from five patients with *A*. *baumannii* infection. *** represents p < 0.001, “ns” represents p > 0.05.

To further investigate the prevalence and commonalities of pathogen migration, we performed sequence alignment analysis. After rigorous quality filtering and sequence assembly, we successfully identified the migration patterns of *A. baumannii* across BALF, sputum, and OPS from five patients. Our analysis revealed that the proportion of identical bases among the three sample types from a single patient was significantly higher than that observed between different patients (**Figure 4A**), indicating a high degree of genetic similarity within individuals. which may reflect the individual-specific characteristics of microbes. Similar results were also observed in *Enterococcus faecium* and *P. aeruginosa* (**Supplementary Figure S7**).

SNP analysis of *A. baumannii* revealed distinct clustering patterns within the same patient, while samples from different patients showed clear separation (**Figure 4B**). Multiple sequence alignment further highlighted individual-specific SNP profiles. For example, in region 1 (**Figure 4C**), Patient 1 and 2 exhibited unique SNP patterns distinct from the other three patients. In region 2, *A. baumannii* sequences from Patient 1 and 2 were consistent across different sample types within the same patient but differed between patients. Similarly, in regions 3 and 4, we observed distinct *A. baumannii* sequences among Patient 3, 4, and 5. These findings underscore the heterogeneity in the migration patterns of *A. baumannii* and reveal the existence of personalized microbial migration signatures during infection.

### Association between pathogen migration and clinical outcomes

To evaluate the clinical relevance of pathogen migration, we compared disease severity, prognosis, and outcomes between infected patients with causative pathogen migration and those without. Among the 56 infected patients, 30 had causative pathogen migration, while 26 did not. The migration group had a significantly higher proportion of severe cases (93.33%, 28/30) compared to non-migration group (61.54%, 16/26) (*p* < 0.05). After appropriate treatment, the proportion of patients with improved prognosis was slightly lower in the migration group (56.67%, 17/30) compared to the non-migration group (69.23%, 18/26), but this difference was not statistically significant (*p* > 0.05). After excluding 4 lost-to-follow-up cases (3 from the migration group and 1 from the non-migration group), the survival rate in the migration group (55.56%, 15/27) was also slightly lower than that in the non-migration group (68.00%, 17/25) (*p* > 0.05).

## Discussion

This study provides novel insights into the dynamics of respiratory microbiome composition and pathogen migration patterns in patients with suspected LRTIs. Our findings reveal that microbial communities maintain remarkable stability within individuals across different respiratory tract sites, while exhibiting significant inter-individual variations—underscoring the highly personalized nature of respiratory microbiomes. Through comprehensive analysis of microbial migration, we identified that 11.53% of common pathogens in infected patients displayed evidence of translocation, a proportion that increased substantially (51.45%) for clinically confirmed causative agents. These migration patterns carry important implications for understanding disease pathogenesis and may inform the development of more precise diagnostic strategies. Notably, we observed an association between pathogen migration and clinical severity, suggesting that microbial translocation plays a critical role in disease progression and intra-respiratory dissemination.

Studies investigating the microbiome of various respiratory diseases have identified microbial signatures that are associated with the diagnosis, severity, and prognosis (Zhou et al., 2019; Opron et al., 2021; Sulaiman et al., 2021). Previous research has also demonstrated that microbiomes in the gut, oral cavity, nasal cavity, and skin exhibit distinct individualized characteristics, maintaining relative stability even during disease episodes (Zhou et al., 2024). Similarly, our study revealed individualization and stability of respiratory microbiome profiles, regardless of infection status. However, we observed significant differences between infected and non-infected patients, with LRTI patients showing reduced microbial diversity and distinct taxonomic profiles dominated by pathogens like *P. aeruginosa*, compared to the *R. mucilaginosa*-dominated microbiomes in non-infected individuals.

The most striking finding of our study relates to pathogen migration patterns using advanced Bayesian source tracking. By optimizing sequence alignment methods, we were able to trace the origins of specific migrating pathogens and confirm their strain-level identity. To our knowledge, this is the first study to accurately characterize pathogen migration patterns in the respiratory tract. We found that while overall microbial migration rates were low (1.92% in infected patients), pathogenic species showed significantly higher migration propensity (11.53%), clinically confirmed causative pathogens exhibited the highest migration rate (51.45%). Additionally, over 80% of migrated causative pathogens displayed bidirectional migration patterns. The observed migration patterns support the validity of less invasive URT sampling (e.g., OPS) for detecting lower respiratory pathogens, particularly in patients who cannot tolerate bronchoscopy. In fact, there are already studies focusing on the development of non-invasive pathogen detection methods. For example, tuberculosis is a typical LRTI, but many studies have begun to evaluate the performance of oral swabs for tuberculosis detection (Wood et al., 2015; Church et al., 2024; Steadman et al., 2024). Our study further provides theoretical basis for expanding such research to include more pathogenic microorganisms.

In addition, the high frequency of bidirectional migration suggests that nosocomial LRTIs may often originate from upper respiratory tract colonization, highlighting the potential value of URT surveillance in high-risk hospitalized patients (Martin-Loeches et al., 2023). The pathogen migration phenomenon discovered in this study suggests that some of these cases are likely to be caused by pathogenic microbes in aerosols entering the URT during hospitalization and then migrating to the LRT, leading to infection. Therefore, periodic collection of URT samples for pathogen monitoring may have a certain preventive effect on nosocomial LRTI infections.

The strong association between pathogen migration capability and disease severity suggests that microbial translocation may be an important virulence mechanism in respiratory pathogens. The association between migration capability and disease severity identifies microbial translocation as a potential therapeutic target in respiratory infections.

While this study provides valuable insights into microbial migration and its clinical implications, several limitations should be acknowledged. First, the single-center design and relatively small sample size, particularly for the non-infected group, may limit the generalizability of our findings. Second, the timing of sample collection, which occurred after patients had already been infected for some time, may have influenced our ability to determine the directionality and temporal dynamics of pathogen migration. Our findings on pathogen migration were derived from single timepoint sampling after infection establishment, which prevented direct observation of the migration process itself. Without longitudinal sampling across different disease stages, we could not determine whether pathogen migration occurred before symptom onset, during disease progression, or as a consequence of infection. Future studies employing serial sampling from symptom onset through treatment resolution could map the temporal progression of pathogen migration, identify critical transition points in disease progression, and potentially uncover novel therapeutic targets.

## Conclusions

Our study provides the first comprehensive characterization of pathogen migration patterns throughout the respiratory tract. It provides a direction for understanding the potential mechanism of maintaining microbiome stability at different sites in the respiratory tract. The demonstrated link between microbial migration capability and clinical disease severity offers new insights into respiratory disease pathogenesis and identifies potential avenues for improved diagnosis and treatment strategies. These findings underscore the importance of considering microbial translocation dynamics in both clinical management and future research on respiratory infections.

## Data Availability

The datasets presented in this study can be found in online repositories. The names of the repository/repositories and accession number(s) can be found below: http://ngdc.cncb.ac.cn, PRJCA021452.

## Supplementary material

Supplementary Figure 1 Comparative Analysis of Alpha Diversity Metrics in Respiratory Samples. **(A)** Assessment of Simpson, Chao1, and ACE indices of alpha diversity in BALF samples from patients with infection (red) and those without (blue). **(B)** Similar comparison conducted on sputum samples. **(C)** Similar comparison conducted on OPS samples. Statistical significance is indicated by asterisk markers, with *, **, and *** corresponding to *p* < 0.05, < 0.01, and < 0.001, respectively.

Supplementary Figure 2 Sample-Type Specific Alpha Diversity Indices in Infected and Non-Infected Patients. **(A)** Examination of Simpson, Chao1, and ACE indices among three types of respiratory samples from patients with infection. **(B)** Corresponding analysis for patients without infection. Significant differences are denoted by asterisk markers.

Supplementary Figure 3 Pairwise Bray-Curtis Distance Analysis. The pairwise Bray-Curtis distance is calculated to compare microbial community dissimilarity between different sample types within the same individual. Additionally, the Bray-Curtis distance is computed among patients for the same sample type, with pairwise comparisons made between these groups. Significant variations are highlighted with asterisk markers.

Supplementary Figure 4 Histogram of pathway abundance and stamp analysis among BALF, Sputum and OPS groups.

Supplementary Figure 5 Histogram of pathway abundance and stamp analysis between infection and non-infection groups in OPS **(A)**, Sputum **(B)** and BALF **(C)**.

Supplementary Figure 6 Enumeration of Microbes Detected Across Respiratory Sample Types. A quantitative representation of the number of distinct microbial entities identified in BALF, sputum, and OPS samples.

Supplementary Figure 7 Sequence alignment analysis of migrated pathogens. Stacked barplot illustrating the distribution of identical and differing bases when comparing samples from the same patient, two different patients, or three different patients. Significant variations are denoted by asterisk markers.
