# Supplementary figures and images for: Investigation of dynamic microbial migration patterns in the respiratory tract
Source: Front Cell Infect Microbiol. 2025 Apr 22;15:1542562. doi: 10.3389/fcimb.2025.1542562 (PMC12052712; doi:10.3389/fcimb.2025.1542562)

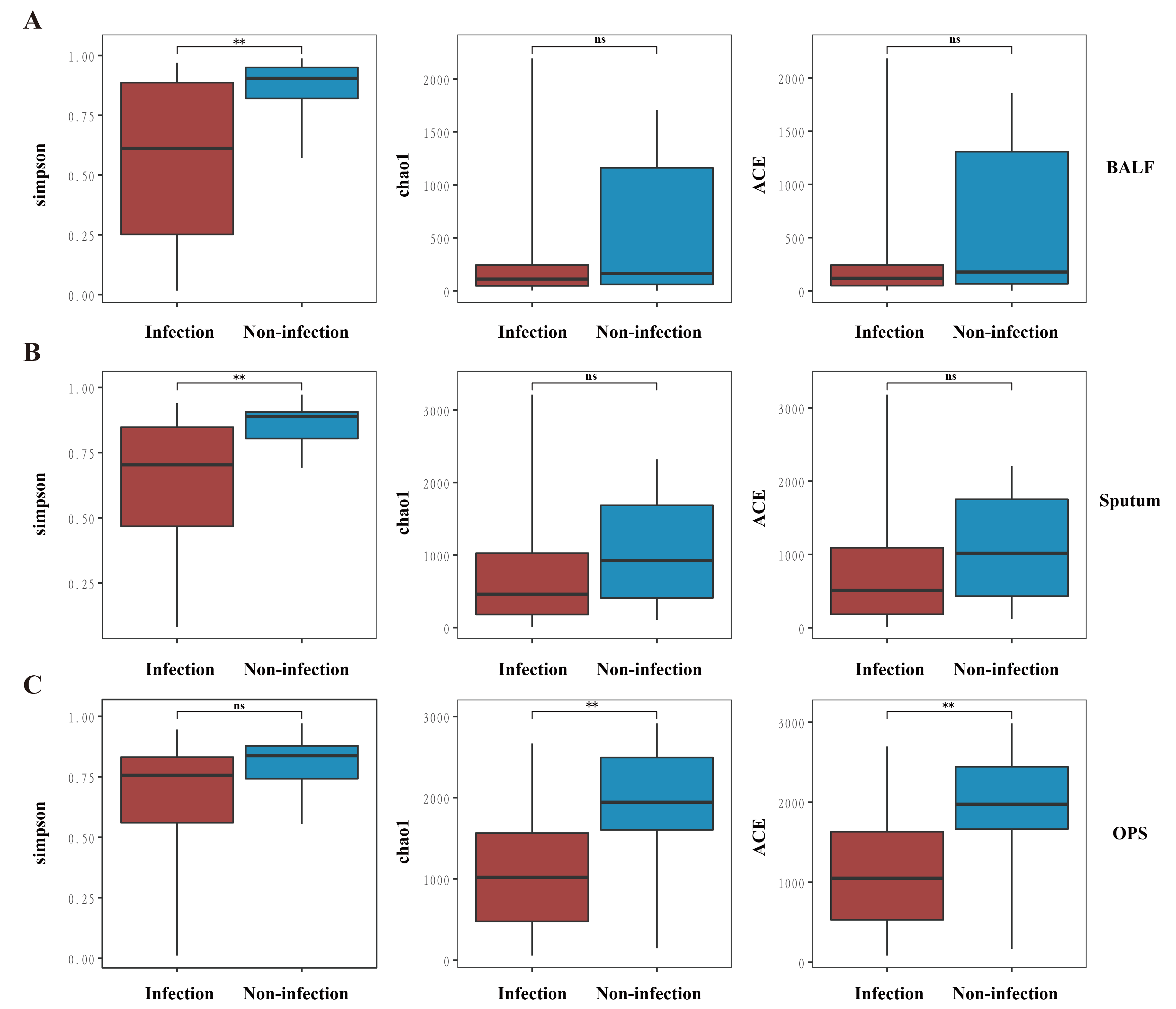

Supplement: Supplementary Figure 1 — Comparative Analysis of Alpha Diversity Metrics in Respiratory Samples. (A) Assessment of Simpson, Chao1, and ACE indices of alpha diversity in BALF samples from patients with infection (red) and those without (blue). (B) Similar comparison conducted on sputum samples. (C) Similar comparison conducted on OPS samples. Statistical significance is indicated by asterisk markers, with *, **, and *** corresponding to p < 0.05, < 0.01, and < 0.001, respectively. [file Image1.tif]

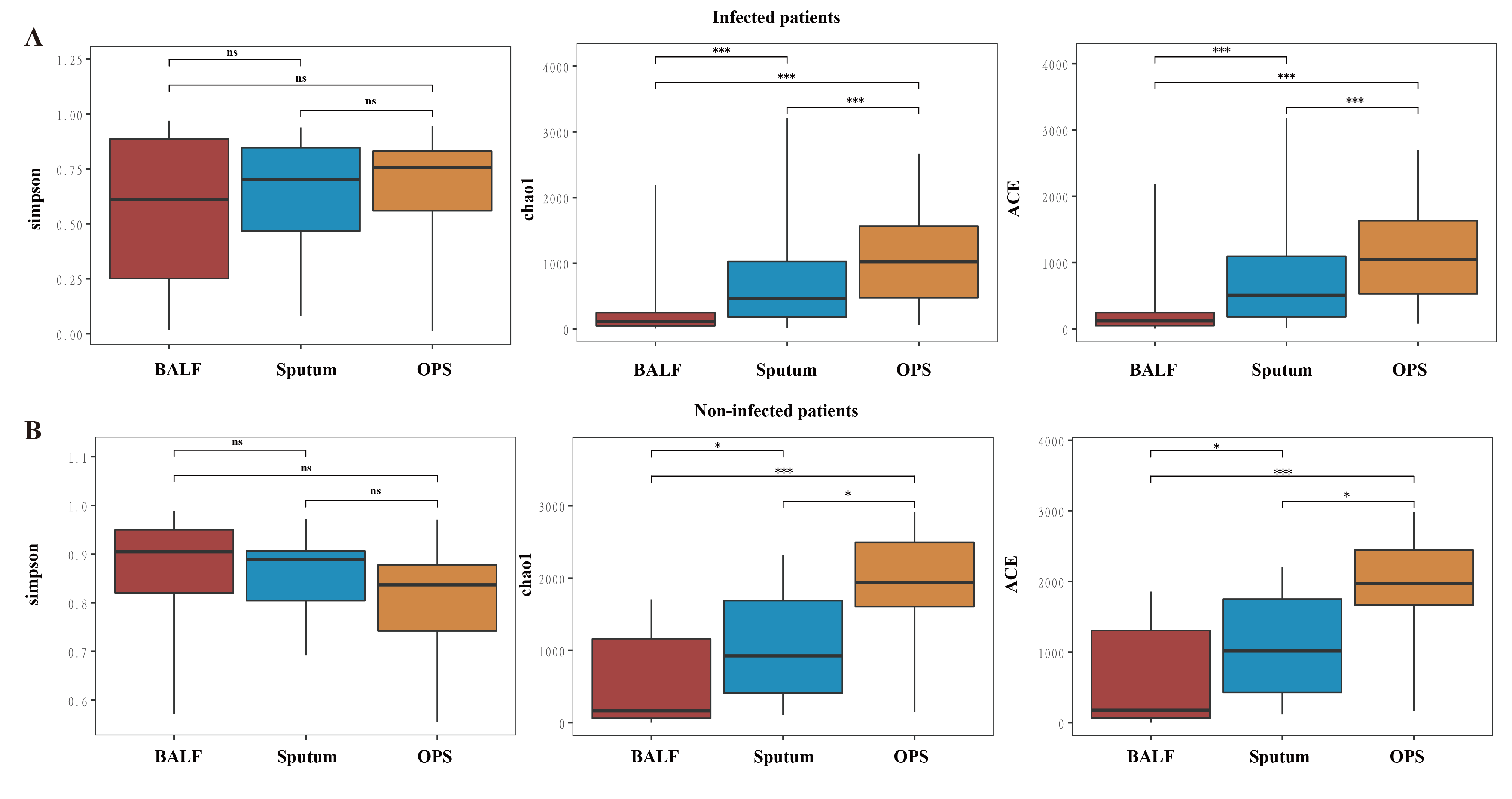

Supplement: Supplementary Figure 2 — Sample-Type Specific Alpha Diversity Indices in Infected and Non-Infected Patients. (A) Examination of Simpson, Chao1, and ACE indices among three types of respiratory samples from patients with infection. (B) Corresponding analysis for patients without infection. Significant differences are denoted by asterisk markers. [file Image2.tif]

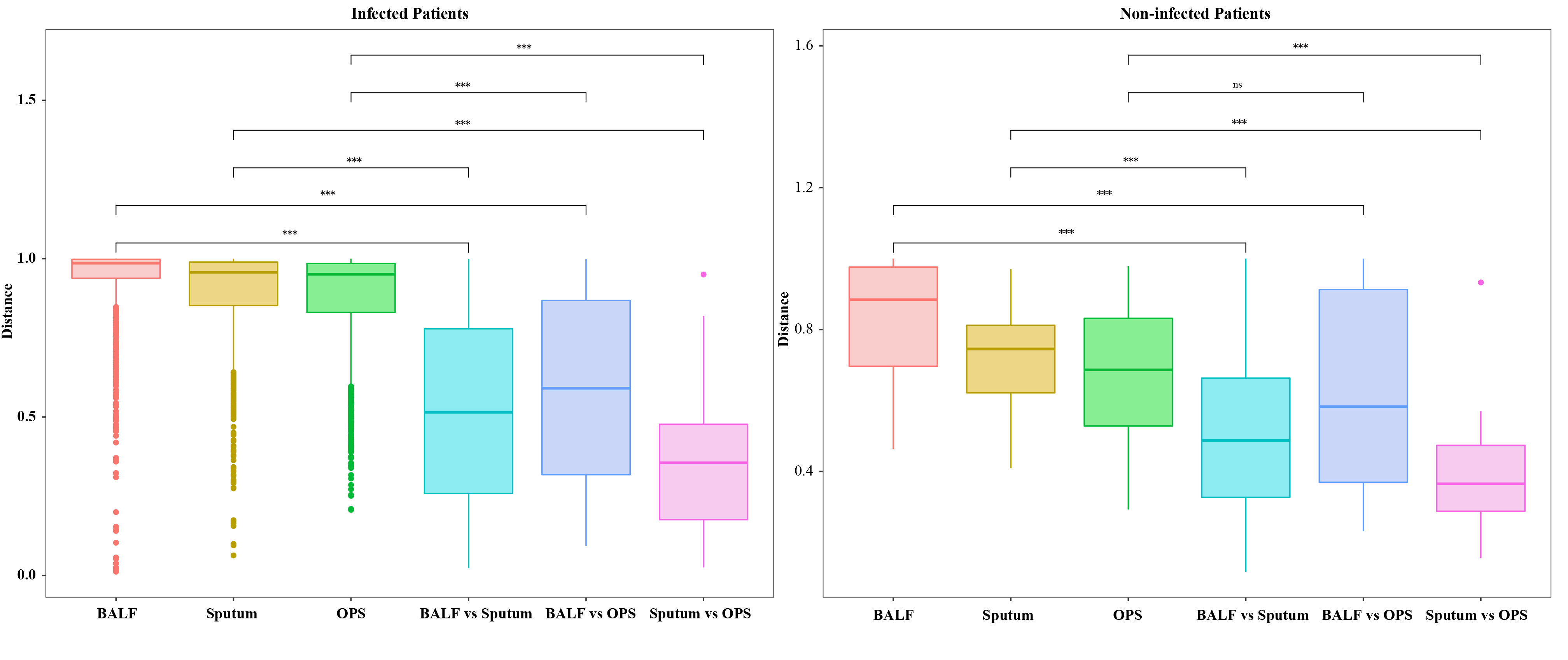

Supplement: Supplementary Figure 3 — Pairwise Bray-Curtis Distance Analysis. The pairwise Bray-Curtis distance is calculated to compare microbial community dissimilarity between different sample types within the same individual. Additionally, the Bray-Curtis distance is computed among patients for the same sample type, with pairwise comparisons made between these groups. Significant variations are highlighted with asterisk markers. [file Image3.tif]

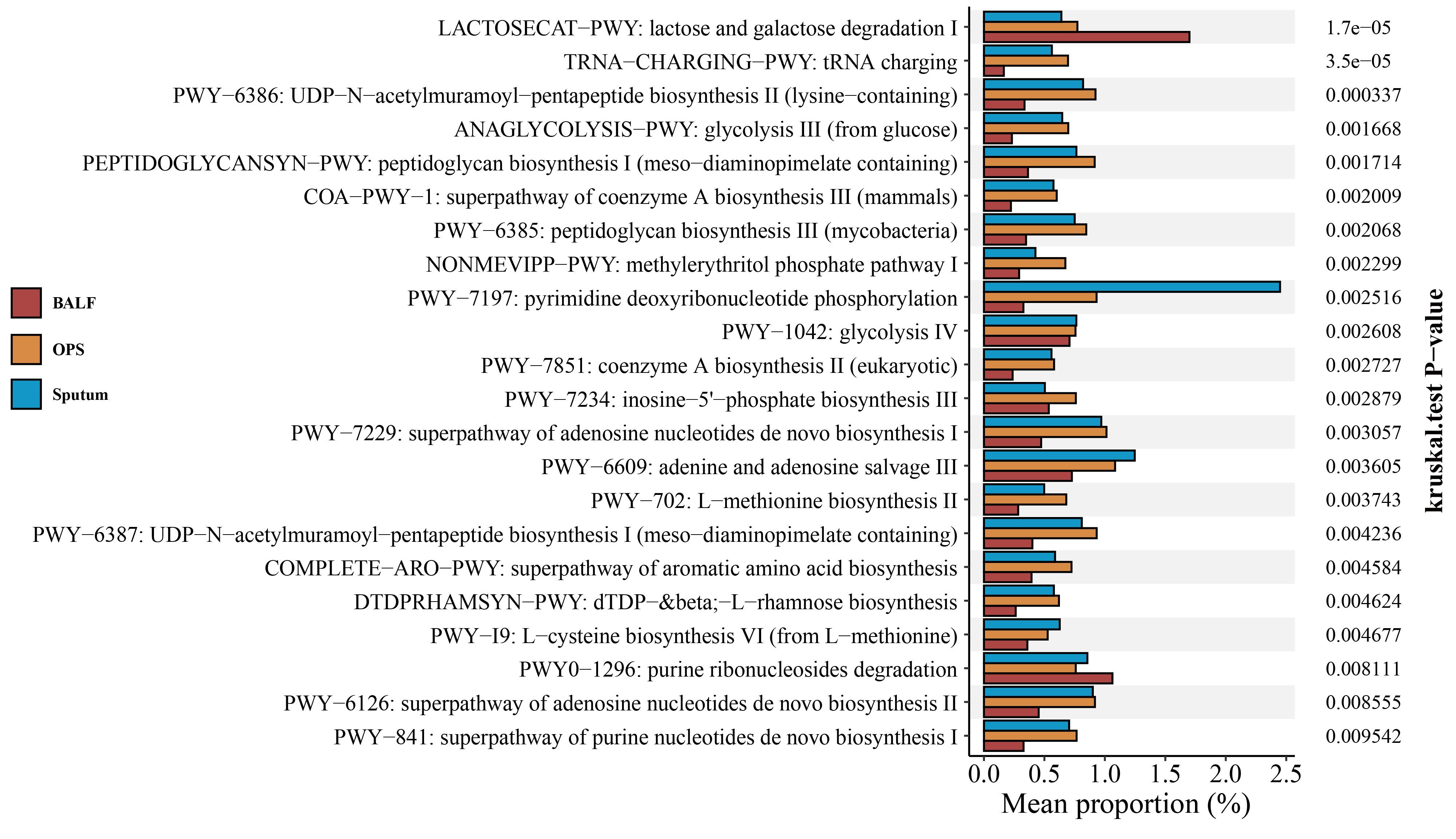

Supplement: Supplementary Figure 4 — Histogram of pathway abundance and stamp analysis among BALF, Sputum and OPS groups. [file Image4.tif]

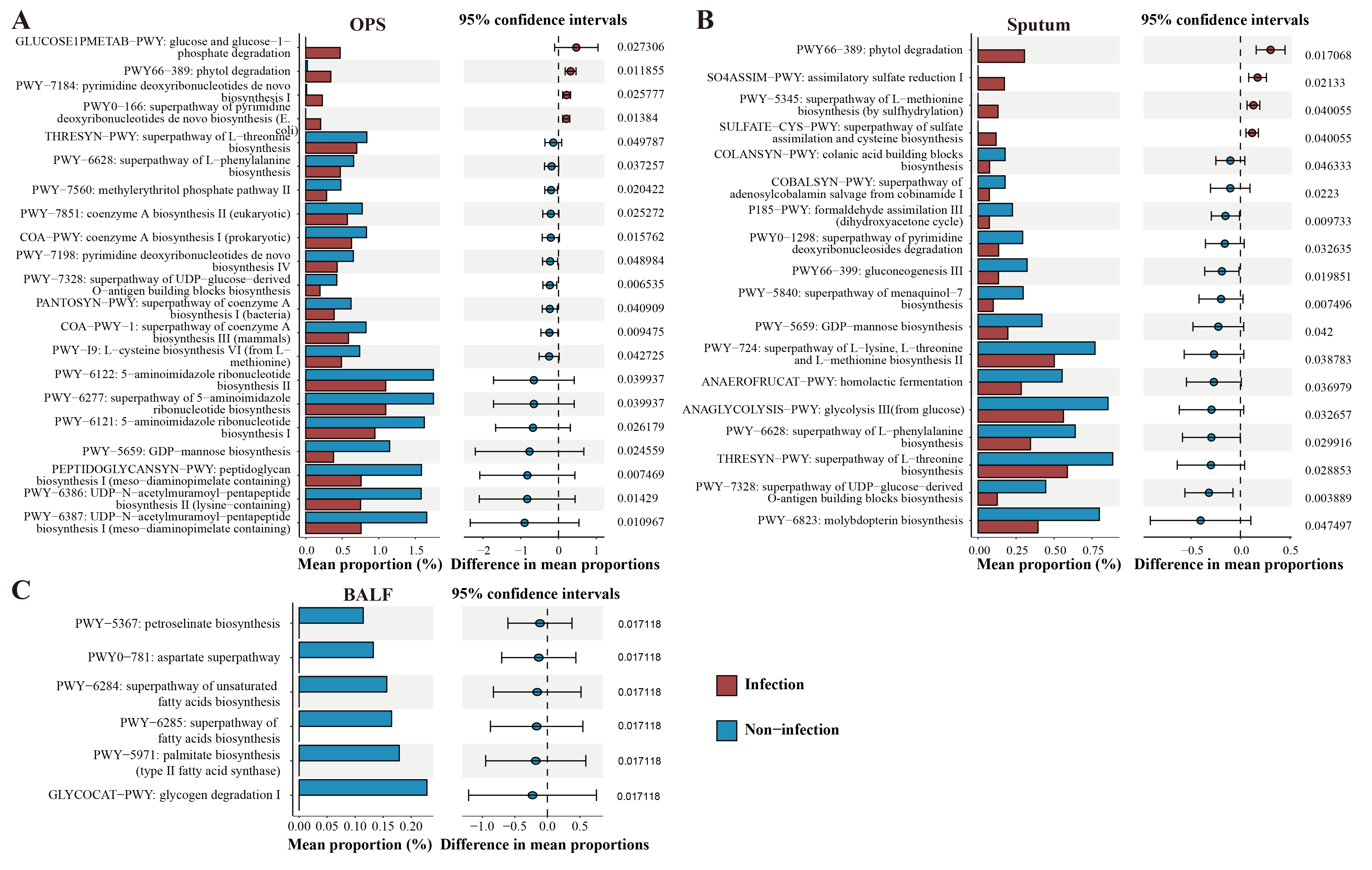

Supplement: Supplementary Figure 5 — Histogram of pathway abundance and stamp analysis between infection and non-infection groups in OPS (A), Sputum (B) and BALF (C). [file Image5.tif]

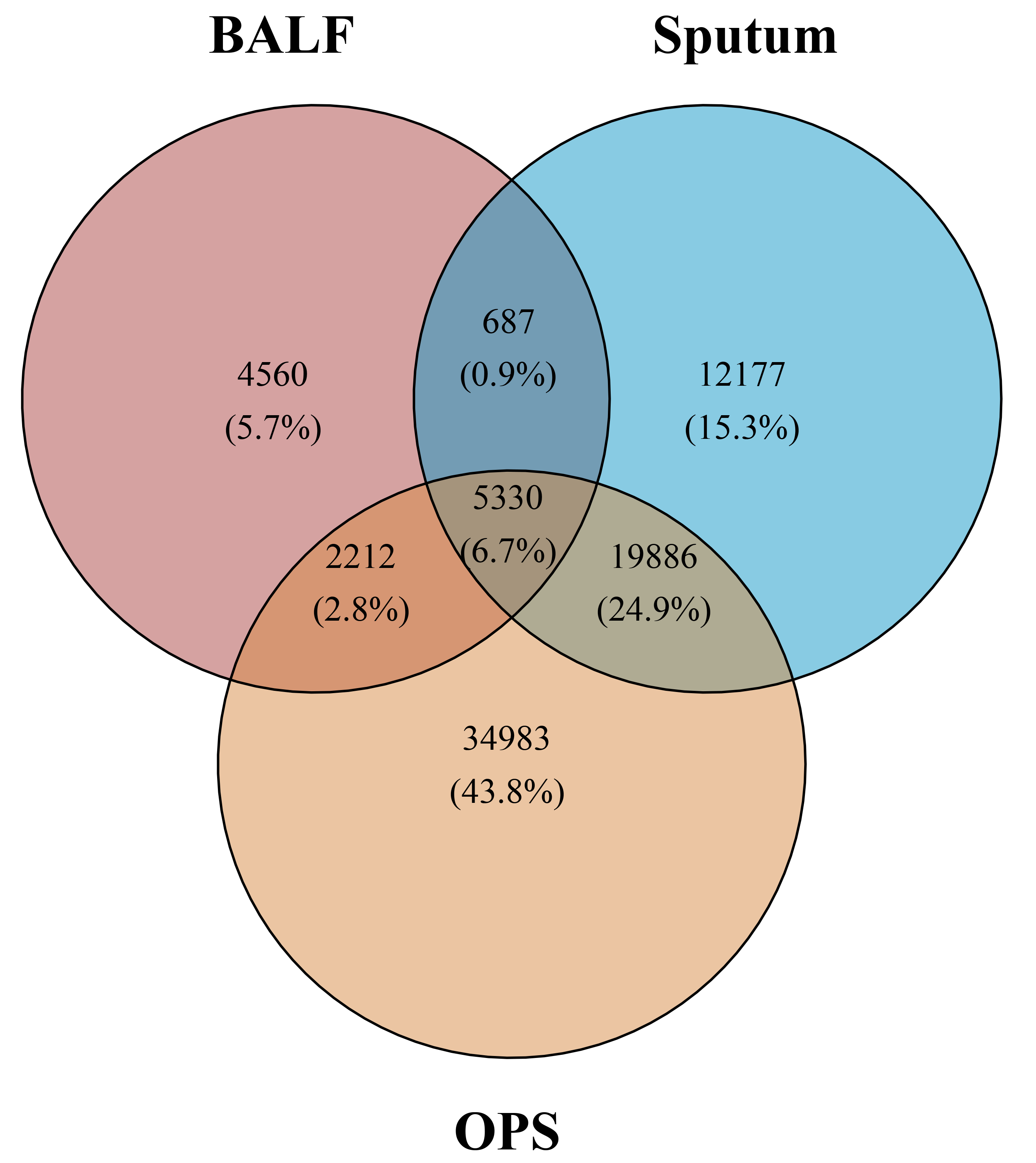

Supplement: Supplementary Figure 6 — Enumeration of Microbes Detected Across Respiratory Sample Types. A quantitative representation of the number of distinct microbial entities identified in BALF, sputum, and OPS samples. [file Image6.tif]

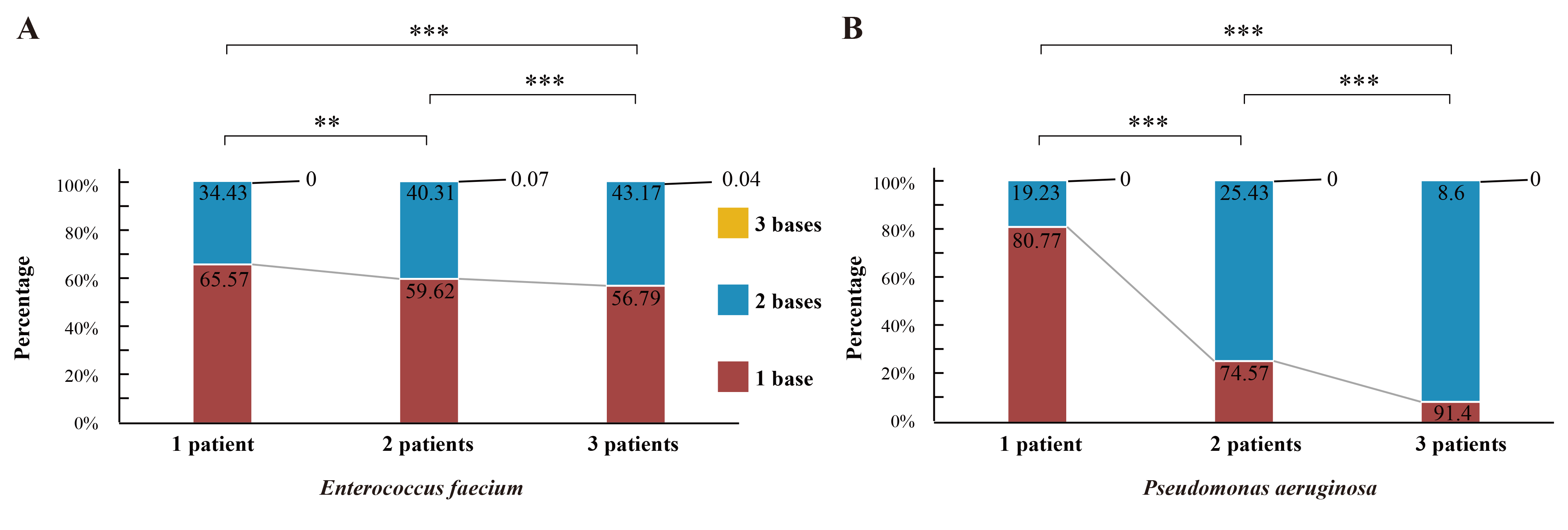

Supplement: Supplementary Figure 7 — Sequence alignment analysis of migrated pathogens. Stacked barplot illustrating the distribution of identical and differing bases when comparing samples from the same patient, two different patients, or three different patients. Significant variations are denoted by asterisk markers. [file Image7.tif]
